# Pathogenicity, tissue tropism and potential vertical transmission of SARSr-CoV-2 in Malayan pangolins

**DOI:** 10.1371/journal.ppat.1011384

**Published:** 2023-05-17

**Authors:** Xianghui Liang, Xiaoyuan Chen, Junqiong Zhai, Xiaobing Li, Xu Zhang, Zhipeng Zhang, Ping Zhang, Xiao Wang, Xinyuan Cui, Hai Wang, Niu Zhou, Zu-Jin Chen, Renwei Su, Fuqing Zhou, Edward C. Holmes, David M. Irwin, Rui-Ai Chen, Qian He, Ya-Jiang Wu, Chen Wang, Xue-Qing Du, Shi-Ming Peng, Wei-Jun Xie, Fen Shan, Wan-Ping Li, Jun-Wei Dai, Xuejuan Shen, Yaoyu Feng, Lihua Xiao, Wu Chen, Yongyi Shen

**Affiliations:** 1 State Key Laboratory for Animal Disease Control and Prevention, Guangdong Laboratory for Lingnan Modern Agriculture, Center for Emerging and Zoonotic Diseases, College of Veterinary Medicine, South China Agricultural University, Guangzhou, China; 2 Guangzhou Zoo & Guangzhou Wildlife Research Center, Guangzhou, China; 3 College of Life Sciences, Longyan University, Longyan, China; 4 Department of Radiology, the First Affiliated Hospital, Nanchang University, Nanchang, China; 5 Sydney Institute for Infectious Diseases, School of Medical Sciences, University of Sydney, Sydney, New South Wales, Australia; 6 Department of Laboratory Medicine and Pathobiology, University of Toronto, Toronto, Canada; 7 Banting and Best Diabetes Centre, University of Toronto, Toronto, Canada; 8 Zhaoqing Branch Center of Guangdong Laboratory for Lingnan Modern Agricultural Science and Technology, Zhaoqing, China; 9 Guangdong Provincial Key Laboratory of Zoonosis Prevention and Control, Guangzhou, China; University of Texas Medical Branch at Galveston, UNITED STATES

## Abstract

Malayan pangolin SARS-CoV-2-related coronavirus (SARSr-CoV-2) is closely related to SARS-CoV-2. However, little is known about its pathogenicity in pangolins. Using CT scans we show that SARSr-CoV-2 positive Malayan pangolins are characterized by bilateral ground-glass opacities in lungs in a similar manner to COVID-19 patients. Histological examination and blood gas tests are indicative of dyspnea. SARSr-CoV-2 infected multiple organs in pangolins, with the lungs the major target, and histological expression data revealed that ACE2 and TMPRSS2 were co-expressed with viral RNA. Transcriptome analysis indicated that virus-positive pangolins were likely to have inadequate interferon responses, with relative greater cytokine and chemokine activity in the lung and spleen. Notably, both viral RNA and viral proteins were detected in three pangolin fetuses, providing initial evidence for vertical virus transmission. In sum, our study outlines the biological framework of SARSr-CoV-2 in pangolins, revealing striking similarities to COVID-19 in humans.

## Author summary

SARS-CoV-2 has caused a severe and ongoing global pandemic. To date, bats and pangolins are the only groups of mammals known carry SARS-CoV-2-related coronaviruses (SARSr-CoV-2). Although the SARSr-CoV-2 detected in confiscated Malayan pangolins from Guangdong, China (PCoV-GD) has lower sequence similarity to SARS-CoV-2 than some bat-derived viruses, its receptor-binding domain within the S protein that binds to host ACE2 receptors for entry into cells is almost identical to that of SARS-CoV-2. Here, we determined the pathogenicity, tissue tropism and transcriptional response of PCoV-GD in deceased adult Malayan pangolins and their fetuses. Notably, virus-positive pangolins showed ground-glass opacities in their lungs similar to COVID-19 pneumonia and blood gas tests that were indicative of dyspnea. We detected viral RNA in multiple pangolin tissues, including liver, intestine, heart, kidney, spleen and skeletal muscle. This is similar to autopsy reports in COVID-19 patients and is associated with multiple organ dysfunction syndrome. The virus-positive pangolins showed an inadequate interferon response. In addition, PCoV-GD was detected in pangolin fetuses, suggesting the potential vertical transmission of the virus *in utero*. In sum, Malayan pangolins infected with a PCoV-GD display a pathology that is strikingly similar to human COVID-19.

## Introduction

After SARS-CoV and MERS-CoV, SARS-CoV-2 is the third coronavirus to cause severe respiratory illness in humans identified in the past two decades. Although characterized by a lower infection fatality ratio [1], SARS-CoV-2 has caused an ongoing and severe global pandemic. SARS-CoV-2 belongs to the genus *Betacoronavirus* of the family *Coronaviridae*, sharing 79.5% overall genome nucleotide sequence identity with SARS-CoV. A better understanding of the pathobiology of SARS-CoV-2 is critical for its control and the development of therapeutics. Several animal models have been developed to help determine the pathogenicity of SARS-CoV-2, including transduced or transgenic mouse models that express human ACE2 [2,3], and macaques that can be infected and recapitulate moderate disease [4,5].

The origin and zoonotic source of SARS-CoV-2 remains uncertain. Bat-derived SARS-CoV-2-related coronavirus (SARSr-CoV-2), such as RaTG13 (from China) and the BANAL isolates (from Laos), are currently the most closely related to the SARS-CoV-2 at the whole genome level, with ~96–97% nucleotide sequence identify [6,7]. Strikingly, Malayan pangolins (*Manis javanica*) were also found to be infected by MERS-CoV-like viruses [8] and at least two SARSr-CoV-2 lineages [9–11]. Although these pangolin SARSr-CoV-2 have lower overall sequence similarity to SARS-CoV-2 than some bat-derived SARSr-CoV-2, the receptor-binding domain of the S protein of the pangolin-CoV-GD (PCoV-GD) is almost identical to that of SARS-CoV-2 [10].

Understanding the biology of SARSr-CoV-2 in their hosts may shed new light on key aspects of coronavirus disease. Previously, the functional comparison of SARS-CoV-2 with closely related pangolin and bat coronaviruses was performed using pseudotyped viruses [12] and spike glycoprotein structures [13,14]. However, the biology of SARSr-CoV-2 in pangolins remains unknown. Herein, we define the pathogenicity, tissue tropism and transcriptional response of PCoV-GD in Malayan pangolins.

## Results

### SARSr-CoV-2 infection in pangolins

We studied 28 adult Malayan pangolins that were confiscated in Guangdong Province between March 2019 to April 2020 and initially sent to Guangzhou wildlife rescue center. Notably, six of these animals were pregnant such that six pangolin fetuses were also available for study. We used the viral S (spike) gene to identify infection with PCoV-GD. This revealed that 14 animals, including six pregnant females, were naturally infected by the virus. All the PCoV-GD positive samples were from the batch confiscated in March 2019. Notably, the S genes from these animals exhibited no differences in the consensus genome sequence. RNA sequencing data enabled the *de novo* assembly of three near complete PCoV-GD genomes that exhibited 99.6–99.9% genetic identity with the PCoV-GD reference genome (EPI_ISL_410721), and 89.8%-90.4% with the SARS-CoV-2 (WHCV). As pangolins are wildlife species, it is possible that they might be infected by viruses other than PCoV-GD which may have in turn impacted pathogenicity. Accordingly, based on the transcriptome data, we found that these pangolins were mainly infected by Sendai virus, pestivirus and parvovirus (S1 Table).

### Pathogenicity of PCoV-GD in Malayan pangolins

**Fig 1:**
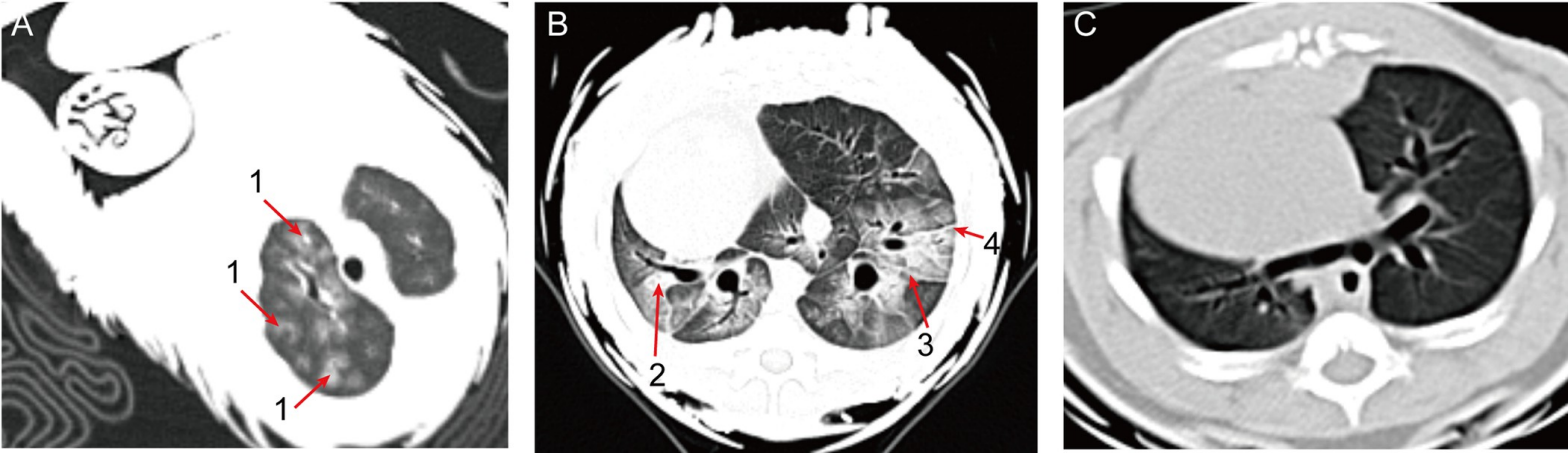
Chest CTs of two PCoV-GD-positive and one virus negative pangolin. (A) Multiple ground-glass opacities in bilateral lungs in one pangolin that was positive to both PCoV-GD and Sendai virus. Arrowheads 1: ground-glass opacities. (B) Bilateral focal consolidation, lobar consolidation, and patchy consolidation were clearly observed in the second PCoV-GD positive pangolin, which was negative for other viruses. Arrowheads 2: air bronchogram; Arrowheads 3: fibrotic streaks; Arrowheads 4: subpleural transparent line. (C) Chest CT of a virus-negative pangolin. This pangolin was negative to PCoV-GD, Sendai virus, pestivirus and parvovirus.

Blood gas tests revealed elevated levels of PCO_2_ (58.2, and 60.25 mmHg), HCO_3_ (29.9, and 34.6 mmol/L), and TCO_2_ (31.5 mmol/L) in the infected animals (S2 Table), indicative of dyspnea. CT scans in one PCoV-GD positive pangolin revealed a diffused bilateral distribution of ground-glass opacities in the lungs (Fig 1A). The other PCoV-GD positive pangolin exhibited air bronchogram, fibrotic streaks and a subpleural transparent line (Fig 1B).

**Fig 2:**
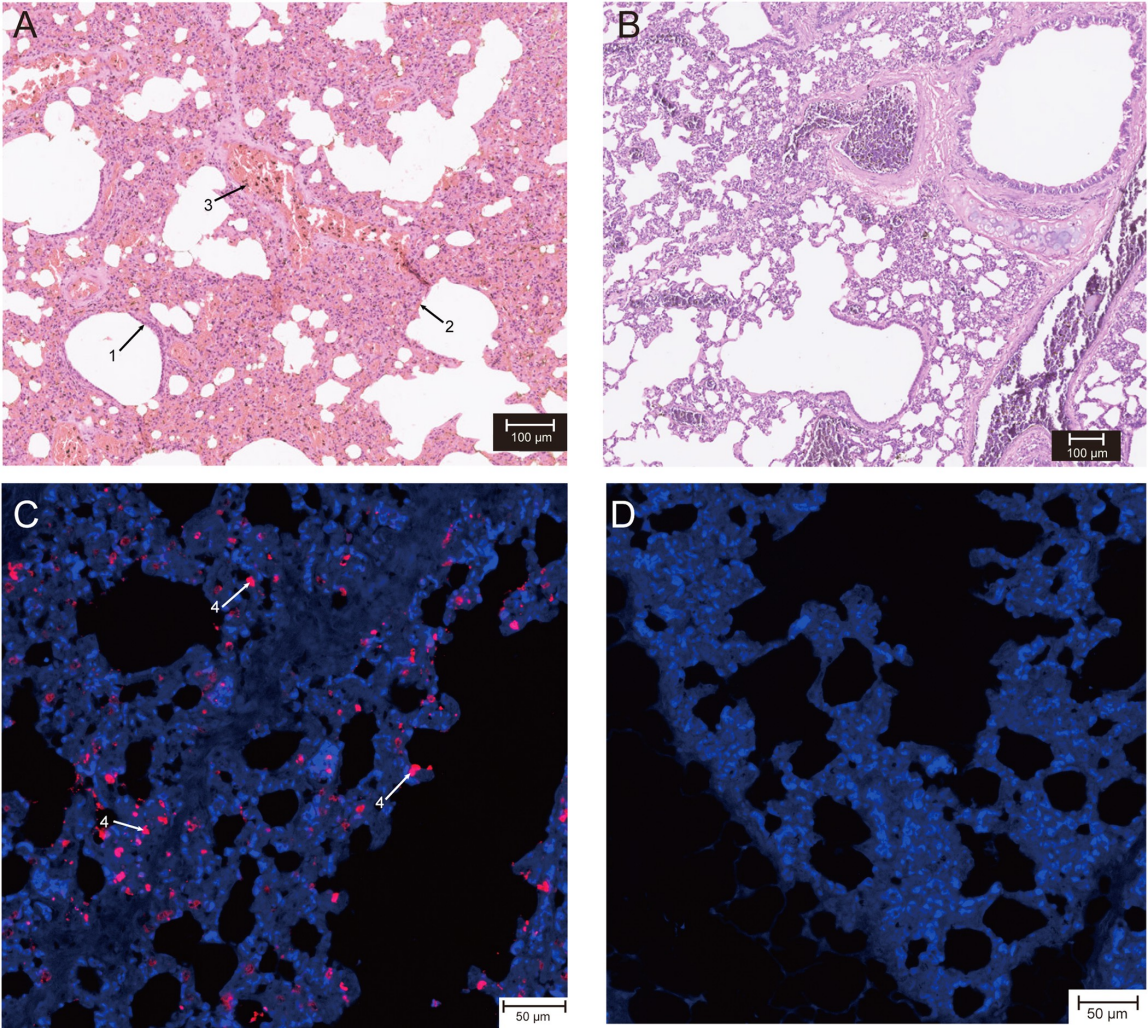
Pathological changes and virus antigen expression in the lungs of one PCoV-GD positive and one negative pangolin. (A) lung sections of a PCoV-GD positive compared to a negative individual (B) were stained by hematoxylin and eosin (H&E). Arrowheads 1, thickened walls of the alveoli; Arrowheads 2, proliferation of alveolar epithelial cell; and red blood cells (Arrowheads 3) are seen. (C-D) Immunofluorescence for virus antigen expression in pneumocytes. (C) nucleocapsid of PCoV-GD (light red, arrowheads 4); (D) negative control. Scale bars of (A) and (B) are 100um, while those in (C) and (D) are 50um. The PCoV-GD positive pangolin (p60) was negative to other viruses. Pangolin tissues were fixed since their necropsy in 2019, hence they were not sufficiently fresh for high quality immunofluorescence, such that the immunofluorescence result was less clear than using fresh tissues.

**Fig 3:**
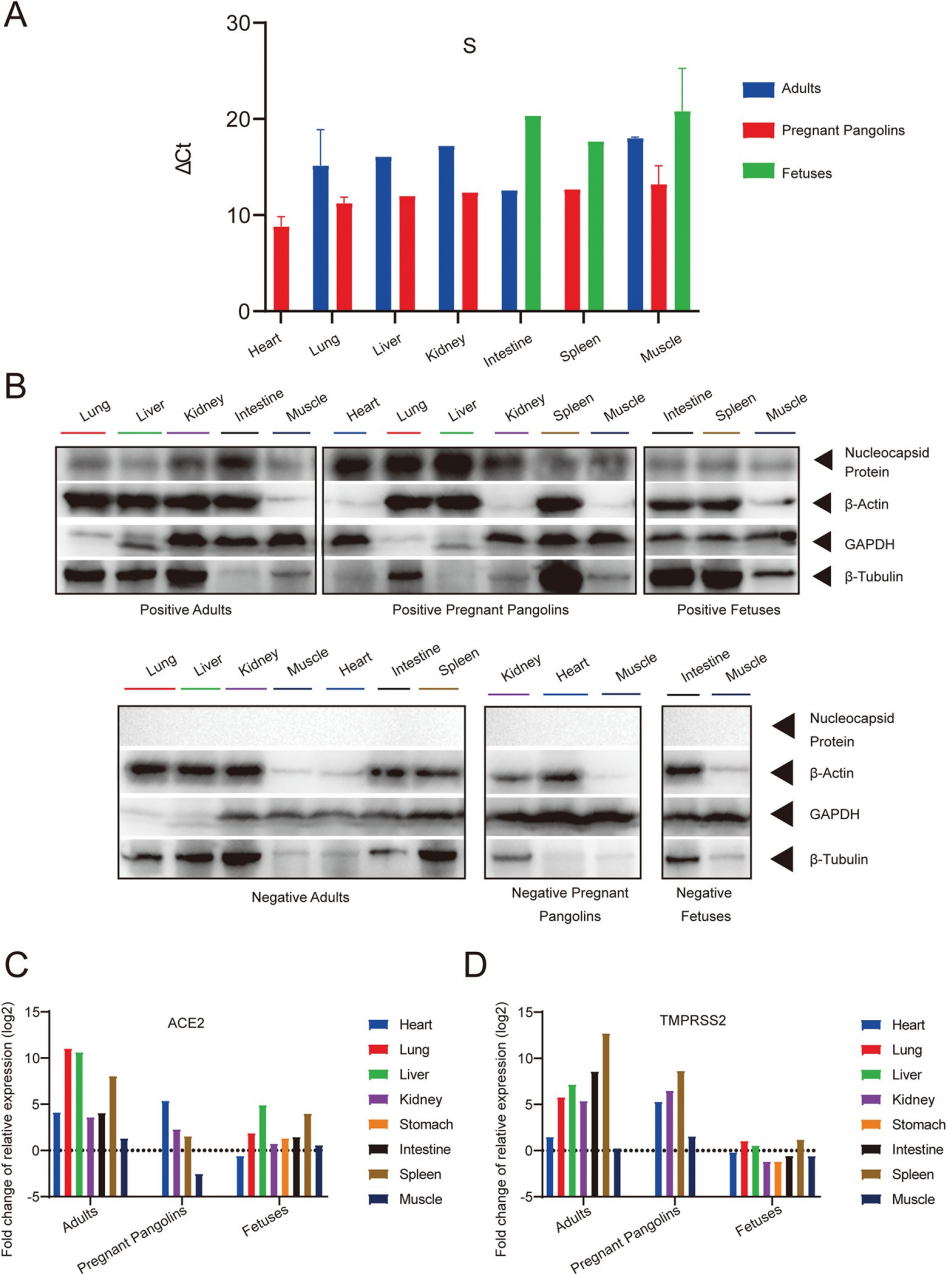
Expression patterns of PCoV-GD and the ACE2 and TMPRSS2 receptor genes in different tissues of adult pangolins, pregnant pangolins and their fetuses. (A) Quantitative real-time PCR (qRT-PCR) of the S gene of PCoV-GD. (B) Western blot of the N protein from the virus-positive tissues. Tissue lysates were subjected to SDS-PAGE and immunoblotting with antisera against SARS-CoV-2 N protein. In this study, we aim to demonstrate the presence of pangolin-CoV N protein in multiple tissues, and it is very difficult to choose a reference protein that expressed in all these tissues. Therefore three reference proteins were used. As the expression of reference proteins inherently varies greatly among tissues, loading control based on the grayscale of the reference proteins is not appropriate for our study. We therefore measured the total protein concentration for each sample, and controlled the consistency of protein loading by adding the same amount of total protein for each sample (30ug). These pangolins died in 2019, prior to the emergence of COVID-19. The importance of these samples was not realized at the time, so that sample collection was incomplete and the numbers of different tissues inconsistent. Fold change of mean values of relative expression of the ACE2 gene (C) and TMPRSS2 gene (D).

Our previous study revealed that four PCoV-GD positive Malayan pangolins had diffuse alveolar damage of varying severity in the lung [10]. To further examine the pathogenicity of the PCoV-GD, lungs from pangolin p60 which was positive to PCoV-GD but negative to other viruses, were stained with hematoxylin and eosin (H&E) for histological examination. Compared with virus-negative pangolins (Fig 2B), the most significant symptom in the lungs was severe interstitial pneumonia, in which the interstitium and the walls of the alveoli were thickened and displayed bronchiectasis (Fig 2A). To ensure these lesions were associated with PCoV-GD, we used immunohistochemistry to detect and localize the nucleocapsid (N protein) of the virus. Several pneumocytes were positive (Fig 2C). qRT-PCR and western blot analysis further supported the presence of PCoV-GD infection in the lungs (Fig 3A and 3B). Necropsy reports of this pangolin revealed hepatomegaly and mucosal injury of intestine (S1 Fig).

### Tissue tropism and potential vertical transmission of PCoV-GD

Necropsy of virus-positive pangolins also revealed multiple organ damage (S1 Fig). We therefore used qRT-PCR to examine the tissue tropism of PCoV-GD. Viral RNA was mainly detected in the lungs, although other organs, including the liver, intestine, heart, kidney, spleen and skeletal muscle of some individuals, also had detectable levels of virus RNA. The six pregnant pangolins had relative lower ΔCt values, and thus higher levels of viral RNA in the lung, liver, kidney and muscle than in adult pangolins. Although pregnant pangolins had relative lower ΔCt values in spleen and muscle compared to fetuses, the different was not statistically significant (Fig 3A and S3 Table). Western blotting further supported the presence of viral infection in these tissues (Fig 3B). PCoV-GD RNA and the virus N protein were detected in the intestine, spleen, and skeletal muscle of three of the six fetuses (Fig 3A and 3B and S3 Table).

Expression of ACE2 and TMPRSS2 was detected in multiple tissues, and the lung, liver, and spleen had the highest levels of ACE2 and TMPRSS expression compared to the other tissues examined (Fig 3C and 3D). PCoV-GD RNA was also detected in the tissues expressing the highest levels of ACE2 and TMPRSS. However, PCoV-GD was also detected in the intestine and kidney, tissues that do not have high expression of these receptor genes in pangolins.

### Host transcriptional response to PCoV-GD

As shown in S1 Table, most PCoV-GD positive pangolins were co-infected by Sendai virus, pestivirus and parvovirus. To reduce the influence of these viruses, individual animals infected by any of these viruses were excluded. After exclusion, two PCoV-GD positive and two negative adult pangolins remained, as well as two PCoV-GD positive and two negative fetuses.

**Fig 4:**
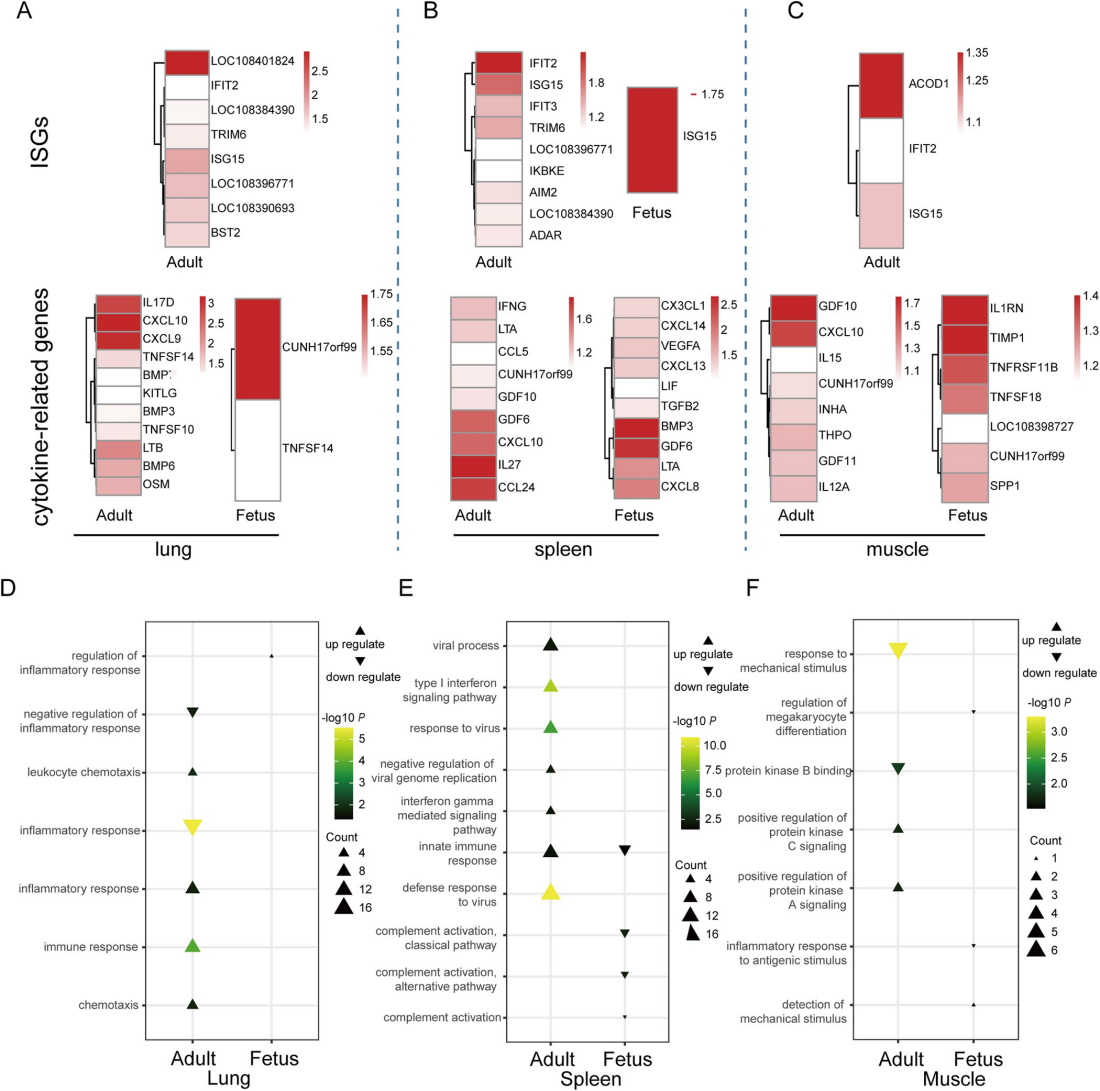
Host transcriptional response to PCoV-GD in the lung, spleen and muscle in adult pangolins and their fetuses. Heatmap of DEGs belonging to ISGs and cytokine-related genes of lung (A), spleen (B) and muscle (C). The graphs show the log_2_(fold change) of DEGs of positive compared with negative animals. The genes included have a log_2_(fold change) of more than 1. Functional enrichment analysis of DEGs of lung (D), spleen (E) and muscle (F).

Up-regulated different expressed genes (DEGs) were associated with the host immune response, such as ISG15, IL17D, CXCL9, CXCL10, LTB and BMP6 in the lung, ISG15, IFIT2, IFIT3, IL27, CCL24 and CXCL10 in spleen, and ISG15, IL12A and CXCL10 in muscle (Fig 4). These DEGs were enriched in the immune response and inflammatory response et al. in the lungs, while the type I interferon signaling pathway, defense response to virus, and innate immune response were up-regulated in the spleens of adult animals (Fig 4D–4F and S4 Table). In the case of the fetuses there were no up-regulated ISGs and very few up-regulated DEGs associated with immune response in lungs. In contrast, ISG15, CXCL8, CXCL13 et al. were up-regulated in the spleen, and IL1RN, TNFRSF11B, TNFSF18 et al. up-regulated in muscle. Functional enrichment analysis of DEGs showed that the fetuses were likely characterized by an inadequate immune response (Fig 4D–4F and S4 Table).

## Discussion

COVID-19 is characterized by a range of symptoms, in most cases including fever, cough, dyspnea, and myalgia [15]. Malayan pangolins that were positive for PCoV-GD exhibited respiratory symptoms such as cough and shortness of breath. Bilateral opacities on x-ray or patchy shadows and ground glass opacities visible in CT scans are the most common features of severe COVID-19 cases [16,17]. Pangolin CT scans showed a diffused bilateral distribution of ground-glass opacities in the lungs of one virus positive pangolin (Fig 1A), similar to early-phase COVID-19 pneumonia [16,17]. The other virus-positive pangolin exhibited air bronchogram, fibrotic streaks and a subpleural transparent line (Fig 1B), similar to advanced COVID-19 disease [18]. Histological examination further revealed the interstitium and the walls of the alveoli were thickened and displayed bronchiectasis, supporting the severe interstitial pneumonia (Fig 2). These features are indicative of dyspnea and consistent with the blood gas tests that revealed elevated levels of PCO_2_, HCO_3_, and TCO_2_ in the infected animals (S2 Table). Hence, pangolins naturally infected with PCoV-GD show similar symptoms and CT features as COVID-19 pneumonia.

The spike protein of SARS-CoV-2 binds to ACE2 receptors for entry into cells, while TMPRSS2 mediates spike protein activation and facilitates viral entry [19–21]. In humans, ACE2 is abundant in the epithelia of the lung, kidney, and small intestine [22]. Although respiratory symptoms dominate the clinical presentation of COVID-19, SARS-CoV-2 is also associated with multiple organ dysfunction syndrome [15], and SARS-CoV-2 RNA is detected in multiple organs [23]. In pangolins, ACE2 and TMPRSS are also expressed in multiple tissues (Fig 3C and 3D), and similar to COVID-19 patients, viral RNA was detected in these tissues (Fig 3A and S3 Table). Hence, these data are suggestive of viral damage to multiple organs (S1 Fig). However, whether the expression levels of ACE2 and TMPRSS2 drive virus tropism needs further study.

The ACE2 receptor is widely expressed in the placenta during pregnancy [24], presenting a theoretical risk of vertical transmission of SARS-CoV and SARS-CoV-2. The risk of SARS-CoV-2 infection in pregnant women and the potential risk of vertical transmission of SARS-CoV-2 are of great concern [25,26]. Although neonatal cases of SARS-CoV-2 infection and antibodies in newborns have been identified [27–29], testing was only performed in newborns such that infection after birth, or the transfer of antibodies from the placenta and breast milk, cannot be excluded. We detected PCoV-GD RNA at a relative higher level in the six virus-positive pregnant pangolins (Fig 3A). This may mean that pregnant animals are more susceptible to the virus, although this will need to be confirmed with a larger sample size. Further, viral RNA and the N protein were detected in three pangolin fetuses (Fig 3A and 3B and S3 Table). Hence, these results are compatible with the vertical transmission of PCoV-GD *in utero*, although this again needs to be confirmed with a larger sample size.

The S genes from these virus-positive pangolins exhibited no changes in the consensus sequence. That the virus genomes across all the animals sampled exhibited 99.6–99.9% genetic identity is indicative of a localized outbreak in these animals due to a single source of infection. As such, it is appropriate to compare their transcriptional response to the PCoV-GD. Interferon induction of ISGs is an essential part of the antiviral response after cellular detection of viral entry into a host cell [30,31]. In a similar manner to SARS-CoV and SARS-CoV-2 [32,33], transcriptome analysis revealed only small significant increases of ISG expression in virus-positive pangolins in all tissues, implying an inadequate interferon response (Fig 4). It is therefore possible that PCoV-GD may inhibit IFN1 by regulating IFN-β synthesis and signaling to avoid the innate immune response in a similar manner to other coronaviruses [34–36]. CXCL10 is associated with severe disease in SARS-CoV [15,33,37]. Similarly, we observed the up-regulation of CXCL10 in the lung, spleen and muscle of adult pangolins. While the fetuses showed inadequate immune responses, especially interferon induction of ISGs in all tissues, this may due to their underdeveloped immune system.

Our study has some limitations. As pangolins are endangered and protected, all their tissues were necessarily collected after their death. In addition, this is not a controlled challenge study as pangolins studied were naturally infected by PCoV-GD. As the pangolins were wild caught, they were infected with additional pathogens, and after co-infected individuals were excluded only a small number of animals were available for study. These caveats might impact analyses of their immune responses.

In sum, although PCoV-GD is genetically distinct from SARS-CoV-2, we show that infected Malayan pangolins exhibit a strikingly similar pathogenicity to human COVID-19. In addition, we show that PCoV-GD was present in pangolin fetuses and was present at high levels in pregnant pangolins, highlighting the potential for virus transmission *in utero*.

## Materials and methods

### Pangolin samples

The Malayan pangolin (*Manis javanica*) is classified as critically endangered in accordance with the International Union for Conservation of Nature (IUCN) Red List of Threatened Species R, and included in Appendix I of the Convention on International Trade in Endangered Species of Wild Fauna and Flora (CITES I). The 28 adult Malayan pangolins, six of which were pregnant, used in this study were confiscated by Customs and the Department of Forestry of Guangdong Province between March 2019 to April 2020. Of these animals, 21 were part of our previous study [10]. These animals were initially sent to Guangzhou wildlife rescue center, during which time some presented with respiratory symptoms. During animal rescue, computed tomographic (CT) scans were performed on two sick animals. For comparison, the CT scan of another pangolin without respiratory symptoms was also performed. Unfortunately, despite exhaustive rescue efforts, all of the pangolins, including the virus-negative ones, died. To determine the cause of their death, veterinarians at the rescue center performed autopsies. Tissues, including lung, liver, spleen, skeletal muscle, kidney, intestine, and heart were collected and stored at -80°C. As most pangolins died in 2019, prior to the emergence of COVID-19, the importance of these samples was not appreciated at the time so that sample collection was incomplete. Available tissue samples are shown in S3 Table. Secondary tissue usage was approved by Guangzhou Wildlife Research Center (permit number: GZZOO20190827C).

### qRT-PCR analysis

PCoV-GD viral RNA, as well as ACE2 and TMPRSS2 transcript levels, were measured by real-time PCR using SYBR Green chemistry. Total RNA was isolated using the QIAamp Viral RNA Mini Kit (Qiagen) according to the kit instructions. 5μg of total RNA was reverse transcribed into cDNA in a 20 μL reaction volume using by PrimeScript IV 1st strand cDNA Synthesis Mix (Takara, Code No. 6215A). For quantitative real-time PCR (qRT-PCR), cDNA was used with ChamQ Universal SYBR qPCR Master Mix (Vazyme, Code No. Q711-02) in a CFX Connect Real-Time System (BIO-RAD). Glyceraldehyde-3-phosphate dehydrogenase (GAPDH) was selected as a host reference gene to normalize the relative viral loads, evaluated by calculating -ΔΔCt values. Each quantitative PCR was performed in triplicate. For PCoV-GD viral RNA detection, a PCoV-GD positive and a negative sample were used as control. The primers used are shown in S5 Table, and are specific such that they do not cross-react with other sequences in the pangolin genome.

### Western blots

Protein was extracted with radio-immuno precipitation assay (RIPA) buffer (50 mM Tris, pH 8.0, 150 mM NaCl, 1.0% NP-40, 0.5% sodium deoxycholate, 0.1% SDS) and a protease inhibitor cocktail. 10mg of total protein was separated on SDS-PAGE and then transferred to PVDF membranes using the Bio-Rad Trans-Blot protein transfer system. The membrane was blocked at room temperature for 90 min with 5% skim milk in 1 × TBST (20 mM Tris–HCl, pH 7.4, 150 mM NaCl, and 0.1% Tween 20). After blocking, the membrane was incubated with SARS-CoV-2 nucleocapsid antibody (kindly provided by Dr. Zhengli Shi, Wuhan Institute of Virology), recombinant anti-GAPDH antibody (Abcam, USA), and recombinant anti-actin antibody (Abcam, USA) at 4°C overnight, washed three times with 1 × TBST, and incubated with corresponding peroxidase-conjugated (Thermo Fisher Scientific) or fluorescently labeled (IRDye 680RD or 800CW: Li-Cor Biosciences) secondary antibody. The membranes were washed three times with 1 × TBST, and then scanned on an Odyssey Infrared Imaging System (Li-Cor Biosciences).

### Histopathology and immunofluorescence

Lungs from six Malayan pangolins, including four positive for PCoV-GD, were used for histopathology and immunofluorescence. Five of these animals (four virus positive and one virus negative) were used in our previous study [10]. As only ten hematoxylin and eosin (H&E) sections for each sample were available in our previous study [10], and without immunofluorescence results, for the current study their tissue blocks were re-sectioned, and histopathology and immunofluorescence were performed. Histopathology of kidneys and livers from two virus-positive pangolins were also performed.

Tissues were fixed in 10% buffered formalin since their necropsy in 2019 and hence were not of sufficient freshness for detailed analysis. They were washed to remove formalin, dehydrated in ascending grades of ethanol, cleared with chloroform, and embedded with molten paraffin wax in a template. The tissue blocks were sectioned with a microtome, and sections were transferred onto grease-free glass slides, deparaffinized, and rehydrated through descending grades of ethanol and distilled water.

Sections were stained with a hematoxylin and eosin staining kit (Baso Diagnostics Inc., Wuhan Servicebio Technology Co., Ltd.). Other sections were incubated in 3% H_2_O_2_ solution in methanol to block endogenous peroxidase activity after dewaxing in xylene and hydrating in different concentrations of ethanol (100%, 95%, 85%, 75% and 50%). Subsequently, sections were incubated in permeabilizing solution containing 1% Triton X-100 (Beijing Solarbio Science & Technology Co., Ltd) in PBS for one hour and then treated with microwaves for antigen retrieval and washed with PBS. Sections were then blocked using 5% BSA at room temperature for one hour and incubated overnight in a humidified chamber with primary antibody against SARS-CoV-2 nucleocapsid (1:500, SinBiological China) at 4°C. Following three washes in PBS, sections were incubated with goat anti-rabbit IgG H&L (HRP) (1,2000, Abcam, USA)in a humidified chamber for 30 min at 37°C and then incubated with Cyanine 3 Tyramide (Cy3 TSA Fluorescence System Kit, Runnerbio Co. Ltd, China), for 30 min at 37°C. After washing five times with PBS, sections were mounted with Antifade Mounting Medium with DAPI (Beijing ZSGB Biotechnology Co., Ltd). Tissue fluorescence was visualized using a fluorescence microscope (OLYMPUS, Tokyo, Japan).

### RNA sequencing

TRIzol (Invitrogen) was used for the extraction of total RNA, with DNA digested with DNase I before library construction. Ribosome RNA (rRNA) was also removed before library preparation. Sequencing libraries were generated using NEBNext Ultra Directional RNA Library Prep Kit for Illumina (NEB, USA) following the manufacturer’s recommendations and index codes were added to attribute the sequences to each sample. Sequencing of the strand-specific libraries was performed on an Illumina NovaSeq 6000 platform, generating 150 bp paired-end reads. Approximately 12 Gb of raw data were generated for each sample (S1 Table). RNA sequencing progress was performed by Novogene (Beijing Novogene Technologies, Beijing, China).

### Bioinformatic analyses

Adaptor and low-quality sequences were trimmed using fastp (v0.19.7) [38]. Viral sequences were counted by mapping the clean reads to the PCoV-GD reference genome (GIASID: EPI_ISL_410721) through BWA-MEM (v0.7.17) [39]. Viral genomes were *de novo* assembled using Megahit (v1.0.3) [40]. For RNA-seq analysis, the Malayan pangolin genome (YNU_ManJav_2.0) was used as the reference host genome [41]. Sequence alignments were performed using the program Hisat2 [42]. Gene counts were summarized using the featureCounts program [43] as part of the Subread package release 2.0.0 (http://subread.sourceforge.net/). Reads mapping and gene counts were performed using the strand-specific pattern for NEB library. Samples with a pangolin genome mapping rate of >70% were used for downstream analysis. Differential expression analysis of two groups was performed using the edgeR package in R [44]. DEGs were characterized for each pair group (|log2 foldchange| > 1, *P* value < 0.01).

To identify pangolin orthologs and make a GO enrichment analysis of different expressed genes (DEGs), the unigenes were conducted by sequence similarity comparisons against the Swiss-Prot database (https://web.expasy.org/groups/swissprot/) with BLASTp (v2.7.1+). A GO enrichment analysis of different expressed genes (DEGs) was performed using clusterProfiler [45] in the R package. Heatmaps of gene expression levels were constructing using pheatmap in R. Dolt plots were constructed using ggplot2 [46]. The heatmap of Type-I IFN responses was constructed on genes with log2 foldchange > 1 belonging to the following GO annotations: GO:0035457, GO:0035458, GO:0035455, GO:0035456, GO:0034340. The heatmap of cytokine activity and chemokine activity was constructed on genes with log2 foldchange > 1 belonging GO annotations for GO: 0005125 and GO: 0008009.

## Supporting information

S1 Fig Photos taken at necropsy from a PCoV-GD positive Malayan. (A) hepatomegaly; (B) mucosal injury of intestine. This pangolin (p60) was positive to PCoV-GD but negative to other viruses.
(TIF)

S1 Table Summary of the pangolin transcriptome data. (DOCX)

S2 Table Blood gas analysis and routine blood tests of pangolins. (DOCX)

S3 Table ΔCt of qRT-PCR of S gene of PCoV-GD, ACE2 and TMPRSS2 genes. (DOCX)

S4 Table Functional annotation clustering for DEGs. (XLSX)

S5 Table Primers used for qRT-PCR. (DOCX)
