# Supplementary figures and images for: Pathogenicity, tissue tropism and potential vertical transmission of SARSr-CoV-2 in Malayan pangolins
Source: PLoS Pathog. 2023 May 17;19(5):e1011384. doi: 10.1371/journal.ppat.1011384 (PMC10228812; doi:10.1371/journal.ppat.1011384)

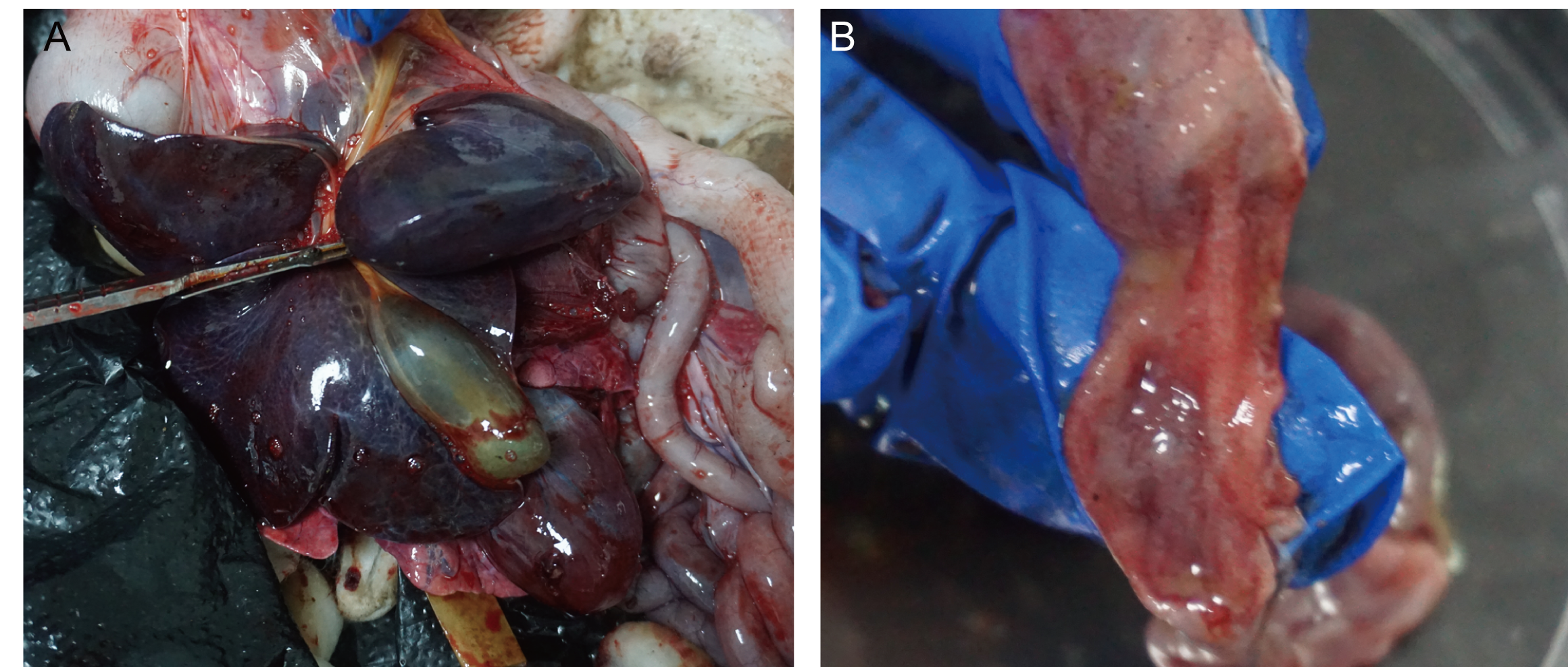

Supplement: S1 Fig — (A) hepatomegaly; (B) mucosal injury of intestine. This pangolin (p60) was positive to PCoV-GD but negative to other viruses. (TIF) [file ppat.1011384.s001.tif]
