# Supplementary material for: Pathogenicity, tissue tropism and potential vertical transmission of SARSr-CoV-2 in Malayan pangolins
Source: PLoS Pathog. 2023 May 17;19(5):e1011384. doi: 10.1371/journal.ppat.1011384 (PMC10228812; doi:10.1371/journal.ppat.1011384)
Supplement: S1 Table — (DOCX) [file ppat.1011384.s002.docx]

S1 Table. Summary of pangolin transcriptome data.

| **Animal IDs** | **Sample IDs** | **Tissues** | **Clean reads** | **Reads mapped to PCoV-GD** | **Reads mapped to Sendai virus** | **Reads mapped to pestivirus** | **Reads mapped to parvovirus** | **Pangolin genome mapping rate** |
| --- | --- | --- | --- | --- | --- | --- | --- | --- |
| P21  Fetus | P21-4-2 | lung | 92128998 | 0 | 0 | 0 | 0 | 94.43%^c^ |
|  | P21-4-6 | intestines | 89084074 | 0 | 0 | 0 | 0 | 91.06%^c^ |
|  | P21-4-7 | spleen | 88396470 | 0 | 0 | 0 | 0 | 93.08%^c^ |
|  | P21-4-9 | muscle | 91804020 | 0 | 0 | 0 | 0 | 90.70%^c^ |
| P38  Fetus | P38-5-2 | lung | 94702454 | 0 | 0 | 0 | 0 | 94.25%^c^ |
|  | P38-5-6 | intestines | 93397918 | 0 | 0 | 0 | 0 | 92.59%^c^ |
|  | P38-5-7 | spleen | 101330570 | 0 | 0 | 0 | 0 | 92.70%^c^ |
|  | P38-5-9 | muscle | 89890414 | 0 | 0 | 0 | 0 | 93.83%^c^ |
| P44  Fetus | P44-6-2 | lung | 92186228 | 0 | 0 | 0 | 0 | 93.63%^c^ |
|  | P44-6-6 | intestines | 91042008 | 0 | 0 | 0 | 0 | 93.13%^c^ |
|  | P44-6-7 | spleen | 89860972 | 1 | 0 | 0 | 0 | 93.53%^c^ |
|  | P44-6-9 | muscle | 88875696 | 1 | 0 | 0 | 0 | 93.20%^c^ |
| P45 (M5)  Fetus | M5f-2 | lung | 90278256 | 0 | 0 | 45965 | 0 | 93.84%^b^ |
|  | M5f-6 | intestines | 106533720 | 0 | 0 | 70238 | 0 | 91.80%^b^ |
|  | M5f-7 | spleen | 90566774 | 0 | 0 | 37210 | 0 | 92.87%^b^ |
|  | M5f-9 | muscle | 90989368 | 0 | 0 | 43029 | 0 | 91.46%^b^ |
| P22  Fetus (P77) | P77-2 | lung | 88845310 | 1 | 0 | 0 | 3430 | 84.77%^b^ |
|  | P77-7 | spleen | 105432226 | 0 | 0 | 0 | 0 | 87.67%^b^ |
|  | P77-6 | intestines | 90799440 | 0 | 0 | 0 | 0 | 85.36%^b^ |
| P79  Fetus | P79-2 | lung | 91370146 | 0 | 0 | 0 | 0 | 91.90%^c^ |
|  | P79-6 | intestines | 91811260 | 0 | 0 | 0 | 0 | 90.89%^c^ |
|  | P79-7 | spleen | 89295216 | 0 | 0 | 0 | 0 | 91.66%^c^ |
|  | P79-9 | muscle | 90657970 | 2 | 0 | 0 | 0 | 91.42%^c^ |
| P21  Pregnant | P21-2 | lung | 108585764 | 80 | 86953 | 868 | 0 | 33.69%^a,b^ |
|  | P21-7 | spleen | 88379884 | 0 | 0 | 28 | 0 | 91.98%^b^ |
|  | P21-9 | muscle | 89429124 | 0 | 0 | 0 | 0 | 84.23%^b^ |
| P22  Pregnant | P22-2 | lung | 98770172 | 1 | 125326 | 800 | 0 | 89.14%^b^ |
|  | P22-7 | spleen | 105466634 | 0 | 0 | 134 | 0 | 86.18%^b^ |
|  | P22-9 | muscle | 100155634 | 0 | 0 | 0 | 0 | 82.17%^b^ |
| P38  Pregnant | P38-2 | lung | 92202160 | 102 | 37035 | 0 | 454 | 88.56%^c^ |
|  | P38-7 | spleen | 92392924 | 0 | 0 | 0 | 0 | 80.89%^c^ |
|  | P38-9 | muscle | 93413456 | 0 | 7 | 0 | 0 | 92.08%^c^ |
| P44  Pregnant | P44-2 | lung | 92600810 | 36 | 6781 | 45861 | 0 | 82.95%^b^ |
|  | P44-7 | spleen | 97396010 | 1 | 0 | 36792 | 0 | 82.45%^b^ |
|  | P44-9 | muscle | 94166460 | 2258 | 32 | 3155 | 0 | 82.45%^b^ |
| P45 (M5)  Pregnant | M5-2 | lung | 83667208 | 28 | 185 | 24213 | 0 | 81.76%^b^ |
|  | M5-9 | muscle | 91492047 | 1289 | 332 | 480 | 9 | 90.72%^b^ |
| P79  Pregnant | P79-9 | muscle | 93828789 | 5314 | 0 | 2 | 0 | 87.39%^b^ |
| P1 | P1-7 | spleen | 113580676 | 0 | 0 | 0 | 0 | 58.76%^a,c^ |
|  | P1-2 | lung | 112577232 | 0 | 0 | 0 | 0 | 56.99%^a,c^ |
|  | P1-9 | muscle | 94802286 | 0 | 0 | 0 | 0 | 67.70%^a,c^ |
| P2 | P2-7 | spleen | 96211922 | 0 | 0 | 0 | 43 | 90.97%^c^ |
|  | P2-9 | muscle | 96747474 | 0 | 0 | 0 | 0 | 88.74%^c^ |
| P11 | P11-2 | lung | 79002516 | 0 | 56 | 0 | 1624 | 90.98%^b^ |
|  | P11-7 | spleen | 100234152 | 0 | 0 | 0 | 278 | 91.09%^b^ |
| P20 | P20-7 | spleen | 101537354 | 0 | 0 | 62 | 0 | 86.67%^c^ |
|  | P20-9 | muscle | 105837878 | 1 | 0 | 0 | 0 | 89.39%^c^ |
| P24 | P24-2 | lung | 94498612 | 0 | 0 | 0 | 16 | 92.49%^c^ |
|  | P24-7 | spleen | 90913580 | 0 | 0 | 0 | 0 | 92.03%^c^ |
|  | P24-9 | muscle | 92163366 | 0 | 0 | 0 | 0 | 89.43%^c^ |
| P25 | P25-2 | lung | 78585396 | 0 | 0 | 28 | 0 | 90.83%^b^ |
|  | P25-7 | spleen | 90252816 | 0 | 0 | 1051 | 0 | 92.07%^b^ |
|  | P25-9 | muscle | 93239184 | 0 | 0 | 0 | 0 | 89.15%^b^ |
| P29 | P29-2 | lung | 93301106 | 1 | 0 | 0 | 0 | 49.81%^a,c^ |
| P36 | P36-2 | lung | 95997962 | 0 | 270516 | 30349 | 0 | 87.80%^b^ |
|  | P36-9 | muscle | 80095618 | 2 | 1396 | 1826 | 0 | 88.59%^b^ |
| P37 | P37-2 | lung | 113134570 | 1 | 0 | 0 | 0 | 60.41%^a,c^ |
|  | P37-7 | spleen | 84728420 | 0 | 0 | 0 | 0 | 54.10%^a,c^ |
| P42 | P42-2 | lung | 91972028 | 0 | 1623098 | 55096 | 138 | 52.60%^a,b^ |
|  | P42-9 | muscle | 117485036 | 21 | 1088 | 50536 | 0 | 74.15%^b^ |
| P47 | P47-2 | lung | 101078552 | 0 | 1308548 | 0 | 772 | 87.52%^b^ |
|  | P47-7 | spleen | 105690874 | 3 | 0 | 0 | 75 | 85.83%^b^ |
|  | P47-9 | muscle | 98545778 | 1 | 101 | 0 | 0 | 92.25%^c^ |
| P56 | P56-2 | lung | 86724130 | 4 | 0 | 29939 | 0 | 77.88%^b^ |
|  | P56-7 | spleen | 79096752 | 0 | 0 | 17268 | 0 | 63.28%^a,b^ |
| P60 | P60-2 | lung | 88648532 | 14 | 0 | 0 | 0 | 82.10%^c^ |
|  | P60-7 | spleen | 88006722 | 0 | 0 | 0 | 0 | 77.09%^c^ |
|  | P60-9 | muscle | 79990510 | 6 | 0 | 0 | 0 | 86.98%^c^ |
| P61 | P61-7 | spleen | 99943752 | 0 | 0 | 0 | 7465 | 87.79%^b^ |
|  | P61-2 | lung | 104404170 | 0 | 0 | 0 | 50743 | 87.79%^b^ |
| P62 | P62-2 | lung | 80683808 | 0 | 0 | 0 | 0 | 72.21%^c^ |
| P63 | P63-9 | muscle | 85429838 | 8 | 0 | 1120 | 0 | 77.63%^b^ |
| P65 | P65-2 | lung | 89630906 | 10 | 0 | 0 | 0 | 84.86%^c^ |
|  | P65-9 | muscle | 89521660 | 0 | 0 | 0 | 0 | 33.50%^a,c^ |
| P70 | P70-2 | lung | 103399481 | 89 | 14 | 81052 | 0 | 38.84%^a,b^ |
|  | P70-9 | muscle | 93370282 | 10 | 0 | 17741 | 0 | 87.53%^b^ |
| P71 | P71-2 | lung | 90390028 | 0 | 0 | 0 | 0 | 89.96%^c^ |
|  | P71-7 | spleen | 103801098 | 0 | 0 | 0 | 0 | 88.56%^c^ |
|  | P71-9 | muscle | 91049328 | 0 | 0 | 0 | 0 | 91.29%^c^ |

Note: ^a^when mapping rates were <70% the data was not be used in subsequent analyses.

^b^data from BioProject accession no. PRJNA901878.

^c^data from BioProject accession no. PRJNA939083.
