# Supplementary material for: Pathogenicity, tissue tropism and potential vertical transmission of SARSr-CoV-2 in Malayan pangolins
Source: PLoS Pathog. 2023 May 17;19(5):e1011384. doi: 10.1371/journal.ppat.1011384 (PMC10228812; doi:10.1371/journal.ppat.1011384)
Supplement: S2 Table — (DOCX) [file ppat.1011384.s003.docx]

S2 Table: Blood gas analysis and routine blood tests of pangolins.

| Individual  Parameters | Malayan pangolin | | | | Dog | Cat |
| --- | --- | --- | --- | --- | --- | --- |
|  | PCoV-GD positive | | PCoV-GD negative | |  |  |
|  | 2019.4.20 | 2019.4.22 | 2018.07.19 | 2018.07.19 |  |  |
| PH | 7.305 | 7.382 | 7.206 | 7.275 | 7.31-7.42^a^ | 7.24-7.40^a^ |
| PCO2 mmHg | 60.25 ↑ | 58.2↑ | 41.6 | 44.5 | 29–42^a^ | 29–42^a^ |
| HCO3 mmol/L | 29.9 ↑ | 34.6↑ | 23.8 | 21.9 | 17–24^a^ | 17–24^a^ |
| BE mmol/L | 3.5 ↑ | 10.0↑ | 2.1 | 1.8 |  |  |
| Hb* g/dL | 13.6 | / | 13.2 | 14.6 | 12-18^b^ | 8-15^b^ |
| PO2 mmHg | 31↓ | / | 54.3 | 65.1 | 85–95^a^ | 85–95^a^ |
| sO2 % | 52 | 97 | 86 | 89 |  |  |
| Na mmol/L | 145 | 144 | 142 | 139 | 142-152^a^ | 146-156^a^ |
| K mmol/L | 4.6 | 4.2 | 4.6 | 5.2 | 3.9-5.1^a^ | 3.6-4.9 ^a^ |
| TCO2 mmol/L | 31.5 ↑ | / | / | / |  |  |
| WBC×10^9^/l | 7.35 | 7.97 | 8.01 | 7.82 | 6.0–15^b^ | 5.5-19.5^b^ |
| RBC×10^12^/l | 6.85 | 7.79 | / | / | 5.0-8.5^b^ | 5.0-10.0^b^ |
| HGB g/l | 150 | 173 | / | / | 120-180^b^ | 80-150^b^ |
| HCT% | 41.15 | 45.56 | 42.3 | 45.6 | 37-55^b^ | 30-45^b^ |
| MCV fl | 60 | 59 | 63.2 | 70.1 | 60-77^b^ | 39-55^b^ |
| MCH pg | 21.9 | 22.2 | 19.95 | 24.21 | 14-25^b^ | 13-20^b^ |
| MCHC g/l | 36.4 | 37.9 | 33.27 | 33.87 | 31-36^b^ | 30-36^b^ |
| PCT | 0.02 | 0.08 | / | / |  |  |
| MPV | 7.1 | 8.1 | / | / | 6.1-13.1^b^ | 12-18^b^ |
| PDWc | 33.1 | 35.3 | / | / |  |  |
| RDWc | 15.9 | 15.3 | / | / | 14-19^b^ | 14-17^b^ |
| LYM×10^9^/l | 1.37 | 1.03 | 1.52 | 1.49 | 1.0-4.8^b^ | 1.5-7.0^b^ |
| MON×10^9^/l | 0.45 | 0.05 | 0.41 | 0.52 | 0.2–1.3^b^ | 0–0.9^b^ |
| NEU×10^9^/l | 5.53 | 6.88 | 4.76 | 5.82 | 2.9–12.0^a^ | 2.5–12.5^a^ |
| LY% | 18.6 | 12.9 | / | / | 12-30^b^ | 20-55^b^ |
| MO% | 6.1 | 0.7 | / | / | 3-9^b^ | 1-4^b^ |
| NE% | 75.2 | 86.4 | / | / | 58–85^b^ | 45–64^b^ |
| ALP U/L | 205 | 190 | 246.61 | 129.18 | 22-114^b^ | 16-65^b^ |
| ALT U/L | 76↓ | 74↓ | 123.52 | 131.59 | 8.2-109^b^ | 25-97^b^ |
| AMY U/L | 371 | 410 | 360 | 228 | 220-1400^b^ | 280-1200^b^ |
| BUN mmol/l | 4.63↓ | 3.20↓ | 6.3 | 5.9 | 2.9-10.0^a^ | 6.8–12.1^a^ |
| CA mmol/l | 2.35 | 2.27 | 2.47 | 2.37 | 2.3–2.9^a^ | 2.2–2.9^a^ |
| PHOS mmol/l | 2.68 | 2.68 | 2.36 | 2.29 | 0.9–1.7^a^ | 1.0–2.0^a^ |
| CRE µmol/l | 8.84 | 26.52 | 71.36 | 56.12 | 44-150^a^ | 80–194^a^ |

Note: ^a^Porter RS, Kaplan JL. The Merck manual of diagnosis and therapy. Merck Sharp & Dohme Corp., 2011.

^b^Sirois M. Laboratory procedures for veterinary technicians. Elsevier Health Sciences, 2014.
