# Supplementary material for: Pathogenicity, tissue tropism and potential vertical transmission of SARSr-CoV-2 in Malayan pangolins
Source: PLoS Pathog. 2023 May 17;19(5):e1011384. doi: 10.1371/journal.ppat.1011384 (PMC10228812; doi:10.1371/journal.ppat.1011384)
Supplement: S3 Table — (DOCX) [file ppat.1011384.s004.docx]

S3 Table. ΔCt of qRT-PCR of S gene of PCoV-GD, ACE2 and TMPRSS2 genes.

| Sample | | | Heart | Lung | Liver | Kidney | Stomach | Intestine | Spleen | Muscle | trachea |
| --- | --- | --- | --- | --- | --- | --- | --- | --- | --- | --- | --- |
| ID | | Gene |  |  |  |  |  |  |  |  |  |
| Mom & fetus  p21 | Mom 1 | S |  | 11.97 |  |  |  |  |  |  |  |
|  |  | ACE2 | 3.16 | 8.13 |  |  |  |  | 5.93 | 10.02 |  |
|  |  | TMRPSS2 | 4.10 | 13.06 |  |  |  |  | 4.22 | 14.25 |  |
|  | Fetus 1 | S |  |  |  |  |  |  |  |  |  |
|  |  | ACE2 | 8.31 | 6.78 | 6.58 | 5.53 | 7.67 | 5.25 | 7.37 | 11.15 |  |
|  |  | TMRPSS2 | 10.19 | 6.06 | 11.77 | 6.51 | 4.81 | 8.78 | 13.05 | 15.39 |  |
| Mom & fetus 2  P22 | Mom 2 | S | 9.93 |  | 11.98 |  |  |  |  |  |  |
|  |  | ACE2 | 0.34 | 14.42 | 2.94 | 1.72 | 0.78 | -0.05 | 9.77 | 10.16 |  |
|  |  | TMRPSS2 | 1.26 | 9.55 | 3.26 | 1.24 | 3.03 | 0.80 | 14.28 | 15.32 |  |
|  | Fetus 2 | S |  |  |  |  |  |  |  |  |  |
|  |  | ACE2 | 10.82 | 10.33 | 10.74 |  |  | 4.82 | 9.30 |  |  |
|  |  | TMRPSS2 | 15.32 | 8.77 | 12.46 |  |  | 5.25 | 11.37 |  |  |
| Mom & fetus 3  p44 | Mom 3 | S | 7.89 | 11.31 |  |  |  |  |  | 11.88 |  |
|  |  | ACE2 | 3.15 | 4.82 |  |  |  |  |  | 8.72 |  |
|  |  | TMRPSS2 | 5.12 | 9.87 |  |  |  |  |  | 15.85 |  |
|  | Fetus 3 | S |  |  |  |  |  |  | 17.67 | 15.98 |  |
|  |  | ACE2 | 8.80 | 5.43 | 6.31 | 5.24 | 5.63 | 4.92 | 2.17 | 10.91 |  |
|  |  | TMRPSS2 | 11.04 | 9.31 | 7.62 | 7.78 | 7.31 | 9.28 | 10.96 | 11.52 |  |
| Mom & fetus 4  P45 | Mom 4 | S | 8.59 | 11.26 |  |  |  |  | 12.70 | 15.41 |  |
|  |  | ACE2 | 3.58 | 2.03 |  |  |  |  | 2.17 | 10.49 |  |
|  |  | TMRPSS2 | 5.03 | 7.75 |  |  |  |  | 8.29 | 10.86 |  |
|  | Fetus 4 | S |  |  |  |  |  |  |  | 24.75 |  |
|  |  | ACE2 | 11.50 | 6.71 | 1.16 | 5.18 | 6.57 | 6.14 | 7.05 | 9.65 |  |
|  |  | TMRPSS2 | 12.12 | 5.65 | 13.56 | 7.02 | 6.72 | 9.64 | 13.47 | 18.37 |  |
| Mom & fetus 5  P38 | Mom 5 | S |  | 10.46 |  | 12.36 |  |  |  |  |  |
|  |  | ACE2 |  | 6.60 |  | 7.00 |  |  | 0.11 | 8.92 |  |
|  |  | TMRPSS2 |  | 7.29 |  | 13.67 |  |  | 7.69 | 16.39 |  |
|  | Fetus 5 | S |  |  |  |  |  |  |  |  |  |
|  |  | ACE2 | 9.64 | 11.45 | 7.15 |  | 6.90 | 7.90 | 7.08 | 9.25 |  |
|  |  | TMRPSS2 | 11.73 | 9.07 | 8.68 |  | 8.59 | 8.90 | 13.31 | 11.31 |  |
| Mom & fetus 6  P79 | Mom 6 | S |  |  |  |  |  |  |  | 12.32 |  |
|  |  | ACE2 |  |  |  |  |  |  |  | 7.19 |  |
|  |  | TMRPSS2 |  |  |  |  |  |  |  | 19.59 |  |
|  | Fetus 6 | S |  |  |  |  |  | 20.35 |  | 21.70 |  |
|  |  | ACE2 | 9.07 | 5.66 | 1.59 | 4.14 | 5.72 | 3.66 | 3.39 | 8.40 |  |
|  |  | TMRPSS2 | 10.23 | 5.01 | 10.20 | 8.72 | 6.71 | 9.29 | 12.58 | 13.69 |  |
| P2 | | S |  |  |  |  |  |  |  |  |  |
|  |  | ACE2 | 7.16 |  |  | 4.51 |  |  | 2.88 | 6.04 |  |
|  |  | TMRPSS2 | 8.44 |  |  | 8.56 |  |  | 14.00 | 14.64 |  |
| P11 | | S |  |  |  |  |  |  |  |  |  |
|  |  | ACE2 | 5.86 | 13.85 |  | 4.38 |  |  | 9.79 |  |  |
|  |  | TMRPSS2 | 6.29 | 7.84 |  | 8.86 |  |  | 13.61 |  |  |
| P16 | | S |  |  |  |  |  |  |  |  |  |
|  |  | ACE2 |  |  |  |  |  |  |  | 9.97 |  |
|  |  | TMRPSS2 |  |  |  |  |  |  |  | 14.4 |  |
| P19 | | S |  |  |  |  |  | 12.59 |  |  |  |
|  |  | ACE2 | -1.29 | -0.03 | -0.61 | 0.47 | -0.09 | 3.54 |  |  |  |
|  |  | TMRPSS2 | 0.60 | 0.05 | 0.68 | -0.28 | 3.62 | -1.70 |  |  |  |
| P20 | | S |  |  |  |  |  |  |  |  |  |
|  |  | ACE2 | 2.18 |  | 12.75 |  |  |  | 10.36 | 12.86 |  |
|  |  | TMRPSS2 | 2.19 |  | 5.77 |  |  |  | 14.52 | 18.01 |  |
| P24 | | S |  |  |  |  |  |  |  |  |  |
|  |  | ACE2 |  | 15.82 | 14.88 | 8.21 |  | 3.26 | 9.62 | 12.27 |  |
|  |  | TMRPSS2 |  | 8.88 | 12.69 | 8.04 |  | 7.03 | 14.84 | 13.01 |  |
| P25 | | S |  |  |  |  |  |  |  |  |  |
|  |  | ACE2 | 6.35 | 14.79 | 13.82 | 7.85 |  | 6.68 | 11.79 | 13.62 |  |
|  |  | TMRPSS2 | 7.17 | 7.53 | 11.06 | 7.71 |  | 8.84 | 13.33 | 13.78 |  |
| P29 | | S |  | 16.92 |  |  |  |  |  |  |  |
|  |  | ACE2 |  | 12.96 |  |  |  |  |  |  |  |
|  |  | TMRPSS2 |  | 8.86 |  |  |  |  |  |  |  |
| P36 | | S |  |  |  |  |  |  |  |  |  |
|  |  | ACE2 | 9.87 | 13.80 | 14.11 |  |  | 4.88 |  | 10.23 |  |
|  |  | TMRPSS2 | 11.31 | 7.57 | 11.36 |  |  | 7.49 |  | 17.30 |  |
| P42 | | S |  |  |  |  |  |  |  |  |  |
|  |  | ACE2 | 7.72 | 15.26 | 11.82 | 5.62 |  |  |  | 10.52 |  |
|  |  | TMRPSS2 | 7.12 | 10.65 | 9.44 | 9.35 |  |  |  | 12.88 |  |
| P47 | | S |  | 19.99 |  | 17.21 |  |  |  |  |  |
|  |  | ACE2 | 12.18 | 13.42 | 9.59 | 5.10 |  |  | 9.68 | 10.01 |  |
|  |  | TMRPSS2 | 13.11 | 6.48 | 8.33 | 5.78 |  |  | 13.76 | 15.04 |  |
| P56 | | S |  |  |  |  |  |  |  |  |  |
|  |  | ACE2 |  | 11.53 | 10.09 | 6.26 |  |  | 9.86 |  |  |
|  |  | TMRPSS2 |  | 7.47 | 11.59 | 7.17 |  |  | 11.13 |  |  |
| P60 | | S |  | 14.06 | 16.08 |  |  |  |  | 17.92 |  |
|  |  | ACE2 |  | 13.89 | 12.35 |  |  |  | 12.10 | 11.06 |  |
|  |  | TMRPSS2 |  | 6.88 | 9.93 |  |  |  | 12.23 | 15.24 |  |
| P61 | | S |  |  |  |  |  |  |  |  |  |
|  |  | ACE2 | 12.59 | 14.37 | 13.37 | 5.54 |  |  | 10.10 |  |  |
|  |  | TMRPSS2 | 14.88 | 9.51 | 13.93 | 6.89 |  |  | 15.58 |  |  |
| P62 | | S |  |  |  |  |  |  |  |  |  |
|  |  | ACE2 | 9.40 | 11.50 | 9.53 | 2.93 |  |  |  |  |  |
|  |  | TMRPSS2 | 10.64 | 7.77 | 8.20 | 4.49 |  |  |  |  |  |
| P63 | | S | 9.14 | 10.21 |  |  |  |  | 7.36 | 10.48 |  |
|  |  | ACE2 | 2.53 | 2.74 |  |  |  |  | 3.39 | 5.46 |  |
|  |  | TMRPSS2 | -6.31 | -7.88 |  |  |  |  | -7.31 | -3.11 |  |
| P65 | | S |  | 17.39 |  |  |  |  |  |  |  |
|  |  | ACE2 | 3.88 | 12.20 |  | 6.03 |  |  |  | 9.79 |  |
|  |  | TMRPSS2 | 3.72 | 8.53 |  | 5.97 |  |  |  | 12.23 |  |
| P70 | | S |  | 9.38 |  |  |  |  |  | 18.09 | 14.62 |
|  |  | ACE2 |  | 6.70 |  |  |  |  |  | 9.14 | 4.35 |
|  |  | TMRPSS2 |  | 8.01 |  |  |  |  |  | 15.53 | 4.01 |
| P71 | | S |  |  |  |  |  |  |  |  |  |
|  |  | ACE2 | 8.62 | 14.10 | 12.06 | 5.91 |  |  | 10.01 | 10.54 |  |
|  |  | TMRPSS2 | 7.82 | 10.72 | 12.07 | 8.89 |  |  | 15.20 | 15.93 |  |
| P80 | | S |  |  |  |  |  |  |  |  |  |
|  |  | ACE2 | 2.72 |  |  |  |  |  |  |  | 0.47 |
|  |  | TMRPSS2 | 0.36 |  |  |  |  |  |  |  | 0.28 |
| P81 | | S |  |  |  |  |  |  |  |  |  |
|  |  | ACE2 | 2.63 |  |  |  |  |  |  |  | 0.58 |
|  |  | TMRPSS2 | 2.38 |  |  |  |  |  |  |  | 0.26 |
| P88 | | S |  | 13.18 |  |  |  |  |  |  |  |
|  |  | ACE2 | 0.44 | 0.72 | 0.43 | 0.31 | 1.26 | -0.67 | 0.44 |  |  |
|  |  | TMRPSS2 | 0.86 | 1.78 | 0.07 | 1.71 | 1.91 | 0.53 | -1.11 |  |  |
