# Supplementary material for: Pathogenicity, tissue tropism and potential vertical transmission of SARSr-CoV-2 in Malayan pangolins
Source: PLoS Pathog. 2023 May 17;19(5):e1011384. doi: 10.1371/journal.ppat.1011384 (PMC10228812; doi:10.1371/journal.ppat.1011384)
Supplement: S5 Table — (DOCX) [file ppat.1011384.s006.docx]

S5 Table. Primers used for qRT-PCR.

| Genes | Primer sequences (5’ to 3’) | Amplicon size (bp) |
| --- | --- | --- |
| GAPDH-qF | AGAGCTGAATGGGAAACTCACTG | 133 |
| GAPDH-qR | TGATGCCTGTTTCACCACCTTC |  |
| Spike-qF | GACAGGTTGATCACTGGCAG | 190 |
| Spike-qR | GTGCTGACTGCGGAAAAGAC |  |
| ACE2-qF | TGACTTCCTGACAGCCCATC | 100 |
| ACE2-qR | ACCCTTCGTTAGCTCCGT |  |
| TMPRSS2-qF | CGCCTCTATGGACCGAACTTCATC | 82 |
| TMPRSS2-qR | TCCAGTCATCTCGGCACACAGG |  |
